# Novel Tools for Adjusting Spatial Variability in the Early Sugarcane Breeding Stage

**DOI:** 10.3389/fpls.2021.749533

**Published:** 2021-11-18

**Authors:** Danilo Eduardo Cursi, Rodrigo Gazaffi, Hermann Paulo Hoffmann, Thiago Luis Brasco, Lucas Rios do Amaral, Durval Dourado Neto

**Affiliations:** ^1^Luiz de Queiroz College of Agriculture, University of São Paulo (ESALQ/USP), Piracicaba, Brazil; ^2^Sugarcane Breeding Program of RIDESA/UFSCar, Araras, Brazil; ^3^Department of Biotechnology, Vegetal and Animal Production, Federal University of São Carlos, Araras, Brazil; ^4^School of Agricultural Engineering, University of Campinas (FEAGRI/UNICAMP), Campinas, Brazil

**Keywords:** proximal sensing, spatial variability, quantitative genetics, geostatistics, envirotyping, *Saccharum officinarum* L. (Poaceae)

## Abstract

The detection of spatial variability in field trials has great potential for accelerating plant breeding progress due to the possibility of better controlling non-genetic variation. Therefore, we aimed to evaluate a digital soil mapping approach and a high-density soil sampling procedure for identifying and adjusting spatial dependence in the early sugarcane breeding stage. Two experiments were conducted in regions with different soil classifications. High-density sampling of soil physical and chemical properties was performed in a regular grid to investigate the structure of spatial variability. Soil apparent electrical conductivity (ECa) was measured in both experimental areas with an EM38-MK2^®^ sensor. In addition, principal component analysis (PCA) was employed to reduce the dimensionality of the physical and chemical soil data sets. After conducting the PCA and obtaining different thematic maps, we determined each experimental plot’s exact position within the field. Tons of cane per hectare (TCH) data for each experiment were obtained and analyzed using mixed linear models. When environmental covariates were considered, a previous forward model selection step was applied to incorporate the variables. The PCA based on high-density soil sampling data captured part of the total variability in the data for Experimental Area 1 and was suggested to be an efficient index to be incorporated as a covariate in the statistical model, reducing the experimental error (residual variation coefficient, CVe). When incorporated into the different statistical models, the ECa information increased the selection accuracy of the experimental genotypes. Therefore, we demonstrate that the genetic parameter increased when both approaches (spatial analysis and environmental covariates) were employed.

## Introduction

Field experiments are essential in plant breeding programs to estimate the genetic parameters and select the best individuals. Plant breeding pipelines incorporate new techniques, such as those derived from genomics, that can support the identification of superior individuals due to genetic factors (Balsalobre et al., 2017; Crossa et al., 2017, 2021; Barreto et al., 2019; Yadav et al., 2020). However, controlling for environmental factors (non-genetic variation) can improve the selection accuracy in field experiments, reducing the experimental error with increasing genetic gain (Crossa et al., 2021; Hoarau et al., 2021; Resende et al., 2021).

Despite advances in phenotyping, genotyping remains superior. The lack of high-throughput data with accessible prices is one of the main reasons that phenotyping routinely impedes acquiring these kinds of data. Additionally, other factors make environmental detailing difficult; for example, Xu (2016) explained that the major environmental conditions are dynamic and can change throughout the crop cycle, and when data are acquired, they are usually considered at the experimental station level and not the plot level (Xu, 2016).

Although the plant breeding techniques that are currently employed are effective, in traditional breeding methods, field experiments are essential for selecting and recommending improved cultivars. High experimental precision is desirable in these experiments and can be obtained with statistical techniques to reduce possible natural variations within experimental fields (Gilmour et al., 1997; Hoarau et al., 2021). When a non-genetic source of variation is modeled, and consequently, isolated from either genetic or residual variations, the accuracy of the model increases, and the comparison between two genotypes is more effective. However, it is often difficult to determine the most appropriate location of the experimental blocks within an experiment when the natural variation in the location is unknown or difficult to measure. This phenomenon is a particular issue in sugarcane breeding programs, wherein large experimental areas (usually more extensive than five hectares) are often needed to assess the performance of hundreds of genotypes. This issue is especially critical in the early stages, in which there are a high number of individuals and restrictions on vegetative material, which makes the use of several basic principles of experimentation, such as repetition, difficult (Cursi et al., 2021).

According to Wei et al. (2015), the assumption of the homogeneity of the location within a repetition or block may not always be valid. This violation can cause inefficient selection within breeding programs, and therefore, reduce genetic gain. In this context, techniques that account for the environmental effects in detail are desired to rationalize the use of inputs and reduce the experimental error in both plot-scale experimentation and field-scale experimentation.

According to Adamchuk et al. (2004), the need for spatial characterizations of both plant and soil factors has led to the emergence of a series of approaches that consider both the use of a high-density soil sampling procedure to better model the effects of spatial variability and the use of proximal field sensing methods for the indirect measurement of soil properties based on optical, electromagnetic, electrochemical, mechanical, airflow and acoustic systems. This ability to identify variations in the field would be highly useful in cultivar selection experiments since the differences identified by the different tools can be employed as an adjustment method while analyzing the genetic potential of each genotype under experimentation.

The objective of this study was to evaluate the efficiency of a digital soil mapping approach and a high-density soil sampling procedure to improve selection in the early sugarcane breeding stage.

## Materials and Methods

### Experimental Areas

The experimental areas considered in this study belong to the Sugarcane Breeding Program of the Federal University of São Carlos (UFSCar), one of the ten federal university members of the Interuniversity Network for the Development of the Sugarcane Industry in Brazil (RIDESA). Here, we considered two experiments, named Experimental Areas 1 and 2 (EA1 and EA2, respectively), corresponding to the first breeding stage, each located in strategic regions of the state of São Paulo, as detailed here. EA1 represents a traditional cultivation region with high soil fertility for sugarcane cultivation, while EA2 represents a region of crop expansion with low soil fertility. Other environmental conditions are available from the RIDESA/UFSCar coverage area, but they are usually considered only in the final assessment trials where a reduced number of genotypes are available. This allows us to account for genotype × environment (G × E) interactions; i.e., the genotypes are tested in multienvironmental trials (MET) across multiple crop-years and seasons. Before the experiments were planted, a high density of soil sampling was performed to determine the effect of the spatial variability. Fertilization and cultural treatments were carried out as recommended for sugarcane.

#### Experimental Area 1

The first experimental area (6.5 ha) is located at the Center for Agricultural Sciences (CCA) at UFSCar, in the city of Araras, state of São Paulo (22°21′25″ S 47°23′03″ W, 650 m). According to the Köppen classification, the climate is characterized as the Cwa mesothermal type, with hot, humid summers and dry winters, an average annual precipitation of 1,300 mm and an average annual temperature of 21.1°C. According to Yoshida and Stolf (2016), the predominant soil in this experimental area is dystrophic red latosol, moderate A, with a clayey texture.

#### Experimental Area 2

The second experimental area (9.7 ha) is located at the Experimental Station of Valparaíso in the northwestern São Paulo state (21°13′20″ S 50°52′00″ W, 460 m). According to the Köppen classification, the region has a tropical climate with a dry season classification (Aw), megathermic, with an average annual precipitation of 1,168 mm and an average annual temperature of 21.9°C. In contrast, this station has predominantly sandy soils, classified as red-yellow podzolic (Dias et al., 1999).

#### Experimental Design

In both areas, the experiments were implemented considering the family structure, which is widely adopted by different sugarcane breeding programs worldwide (Jackson et al., 1995; Barbosa et al., 2004; Zhou et al., 2013; Cursi et al., 2020). Briefly, this structure consists of groups of related individuals from the same crossing (family) who share close genetic information in the same plot, i.e., families are our treatments during data analysis. A possible statistical design is an incomplete block design with replications. In this study, the experimental unit consisted of two rows with a length of 27 m, a spacing of 1.4 m between rows, 54 seedlings per row (spaced at 0.5 m), and a total plot area of 75.6 m^2^. The trial was planned for two replications for each family, but some unbalanced results could be verified. In general, EA1 and EA2 contained 443 families and 432 families, where most of them (418) were common for both places. These families were generated in 2017 at the Flowering and Crossing Station of Serra do Ouro in Muricí-AL (9°14′36″ S 35°50′16″ W, 450–500 m) from the combination of elite parents and, therefore, frequently used by the breeding programs of RIDESA. In total, 63% of the families were obtained from half-sib crosses, and the other 37%, from full-sib crosses. In each experimental block, two commercial varieties (RB855453 and RB867515) were considered as controls. Both experiments were planted in May 2018.

### Soil Sampling Scheme

Before the implementation of the experiments, both areas were georeferenced. Soil sampling was carried out mainly in a regular grid, where samples were collected at equally spaced points and homogeneously distributed throughout the experimental region.

In EA1, 56 points (8.6 samples/ha) were sampled at depths of 0–20 cm. Each sample was composed of six subsamples collected from an average radius of 3 to 5 m around the central point (Supplementary Figure 1A). The same procedures occurred for EA2; however, since EA2 was larger than EA1, 68 sample points (7 samples/ha) were collected (Supplementary Figure 1B).

The soil samples were sent to the soil laboratory of the Department of Natural Resources and Environmental Protection at UFSCar for analysis and determination of chemical (macronutrients) and physical attributes of the soil.

### Apparent Electrical Conductivity Mapping

Thirty days after the experiment was planted, apparent electrical conductivity (ECa) mapping was performed by using the EM38-MK2^®^ sensor (Geonics, Mississauga, Ontario, Canada), which operates with the principle of electromagnetic induction (EMI). In both experimental areas, the sensor, which was connected to a GPS receiver [type L1 (Trimble)], was used to record the geographic coordinates, and a data collector (Juniper Archer Field PC) was used to store information. Additionally, no vehicle was utilized to transport the sensor in either experimental area, i.e., it was manually operated. Readings were collected in all the interrow lines between the crops.

According to the manufacturer’s information, the EM38-MK2^®^ equipment simultaneously provides ECa measurements for 0.75 and 0.375 m in the horizontal dipole orientation. This depth reading was considered for use based on Jung et al. (2005), wherein it was demonstrated that it provides the best relationship between the ECa and the soil properties in layers to 30 cm, which is the depth that was contemplated through soil sampling.

### Data Processing and Analysis

The data were subjected to statistical treatments associated with the use of graphical tools, i.e., histograms and boxplots, to assess the shape and dispersion of the data set.

To reduce or eliminate overlap and to select the most representative forms of data from linear combinations of the variables from the soil analysis, the dimensionality of the data was reduced through principal component analysis (PCA) using R software (R Core Team, 2020). The variables were standardized for mean zero and unity variance. Here, we considered only the first two components since a single biplot could be obtained to summarize the results.

The package geoR version 1.8-1 (Ribeiro and Diggle, 2001) was selected for the geostatistical modeling of the principal components. A theoretical semivariogram was modeled by restricted maximum likelihood, or REML (Kerry and Oliver, 2007), and the tested models were spherical, exponential, and Gaussian. The model’s selection was based on the root mean square error (RMSE), coefficient of determination (R^2^), and mean squared deviation rate (MSDR) *via* cross-validation. Inferences about spatial dependence were based on the classification proposed by Seidel and de Oliveira (2016). This classification was considered to ensure an understanding of the degree of spatial dependence of the different principal components and to identify the intensity of the variability present in the experimental areas.

In this study, we utilized the inverse distance weighting (IDW) statistical interpolator (Shepard, 1968) since this type of interpolation is suitable when dealing with a high-density data set. After obtaining thematic maps from the information of the different principal components, the position and exact location of each experimental plot were determined (Supplementary Figure 2). After the plots on the thematic maps were overlaid, the values of each pixel (interpolation over 0.5 m × 0.5 m pixels) were extracted within each experimental plot, and after undergoing descriptive statistical analysis, the average value was considered and applied as a covariate in the genetic-statistical model, as detailed in equations 1.2 and 1.4.

Regarding the ECa sensor, due to the high density of data collected, all the raw data in both experimental areas were considered and directly plotted on the segmented plots; therefore, no interpolation procedure was required. Afterward, the average ECa of the soil was calculated for all the points obtained within each experimental plot. Each plot-specific mean ECa value was considered and applied as a covariate in the genetic-statistical model, as detailed in equations 1.2 and 1.4.

### Experimental Evaluation and Adjustment of Genetic-Statistical Models

Both experiments were previously evaluated by following the same criteria routinely adopted in the early breeding stages of RIDESA/UFSCar; i.e., each family was measured for cane yield (tons of cane per hectare, TCH) when considering the plant cane stage after 12 months in May 2019. The TCH was obtained through mechanized harvesting and total plot weighing (family) with the support mobile truck-mounted weighing equipment. The estimates were calculated using the following equation: TCH = (TW × 10)/PS, where TW is the total weight of the plot (in kg) and PS is the total plot size in m^2^, i.e., 75.6 m^2^.

We used the linear mixed model approach where TCH was the response variable, and four different statistical models were assumed (from 1.1 to 1.4):


(1.1) $$y_{ij}=\mu +F_{i}+b_{j}+e_{ij},\;\text{where }e_{ij}∼N\left(0,\sigma^{2}\right)$$

$$y_{ij}=\mu +F_{i}+b_{j}+\sum_{\text{k=1}}^{\text{w}}\beta_{k}d_{ij}+{\text{e}}_{ij},$$

(1.2) $$\text{where}\,{\text{e}}_{\text{ij}}∼\text{N}\,\left(0,\sigma^{2}\right)$$

$$y_{ij}=\mu +F_{i}+b_{j}+\varepsilon_{ij},$$

(1.3) $$\text{where}\;{\text{ε}}_{ij}\;∼\;N\left(0,\;\;AR{\left(1\right)}_{u}AR{\left(1\right)}_{v}\sigma^{2}\right)$$

$$y_{ij}=F_{i}+b_{j}+\sum_{\text{k=1}}^{\text{w}}\beta_{k}d_{ij}+{\text{ε}}_{ij},$$

(1.4) $$\text{where}\;{\text{ε}}_{ij}\;∼\;N\left(0,\;\;AR{\left(1\right)}_{u}AR{\left(1\right)}_{v}\sigma^{2}\right)$$

The four models have an intercept (μ), a random effect for i-th family (*F_i_*), a fixed effect for the j-th block (*b_j_*), and an error term.

The error term in models 1.1 and 1.2 assumes homogeneity over all the plots and no associations between them, statistically indicated as **e**_**i***j*_∼*N*(0,σ^2^). For models 1.3 and 1.4, the error term takes the spatial dependence over plots, i.e., the first-order autoregressive structure for rows and columns, or ε_*ij*_∼*N*(0,*AR*(1)_*u*_*AR*(1)_*v*_σ^2^). The comparisons between the absence vs. the presence of spatial dependence (models 1.1 vs. 1.3 and 1.2 vs. 1.4) were performed using the Akaike information criterion (AIC) (Akaike, 1974) and the Bayesian information criteria (BIC) (Schwarz, 1978).

Models 1.2 and 1.4 included the environmental information ($\sum_{k=1}^{w}\beta_{k}\,{\text{d}}_{i\,j}$) captured using either soil sample information or ECa values, where β_**k**_ is the effect of the k-th environmental covariate, and **d**_*ij*_ is the covariate data for the plot containing the i-th family on the j-th block. The covariates inclusion was based on the forward selection approach (Kutner et al., 2004), i.e., (i) each variable was independently tested in the statistical model using the *F*-test; (ii) the covariates were ordered according to the *F*-test; (iii) if the highest was significant under 5%, the covariate was added to the model and the process was repeated to include the next one; otherwise, the process was stopped.

The residual variation coefficient (CVe%) was computed as the proportion of the residual error over the experimental mean for the TCH. The broad-sense heritability (*H*^2^) and the accuracy of selection (AC) were calculated according to the following equations:


$${\text{H}}^{2}=\left(\frac{\sigma_{g}^{2}}{\sigma_{g}^{2}+\frac{\sigma_{e}^{2}}{R}}\right)$$

$$\text{A}\,C=\sqrt{H^{2}}$$

where *H*^2^is the broad-sense heritability at the family mean level; $\sigma_{g}^{2}$ is the variance in genetic effects between families; $\sigma_{e}^{2}$ is the variance in residual effects (environmental); R is the number of repetitions for families; and AC is the accuracy of selection between families.

Before applying mixed models for data analysis, we verified the spatial distribution pattern of the raw data for each plot/family. We selected the R spatstat package version 1.63-3 (Baddeley et al., 2015), which has exploratory data analysis, model adjustment and simulation functionalities.

To perform the statistical analyses, GenStat software (Payne et al., 2011) was used to predict the genotypic values of the treatments (families) considering the different models, and R software (R Core Team, 2020) was selected for the geostatistics and graphical analyses.

## Results

### Exploratory Analysis of Data From EA1

PCA was performed for the 17 physical and chemical variables of the soil. The first two components captured 56.4% of the data variability (Supplementary Figure 3A). The first principal component (PC1) was mainly explained by variables related to soil acidity and base saturation in relation to the cation exchange capacity (CEC), i.e., base saturation, calcium, magnesium, percentage base saturation, percentage aluminum saturation, aluminum, and potential acidity; the only exception was the clay content (Supplementary Figure 3B). The second component (PC2) included a more diverse group of variables, such as pH, phosphorus, silt and the contents of different fractions of sand (total, coarse, and fine sand). The geostatistical analysis on the two principal components showed that, for both fields, PC1 present weak spatial dependence, while PC2 showed strong spatial dependence (Seidel and de Oliveira, 2016; Table 1). This might be explained by the main variables of each PC: most of the variables of PC1 are related to soil chemical properties (acidity), which tend to be more variable within the fields; in contrast, most of the variables of PC2 are related to soil texture (silt and sand contents), which often shows higher spatial dependence.

**TABLE 1:** Parameters of the theoretical models adjusted to the experimental variograms of the data set at a depth of 0–20 cm, Experimental Area 1 (EA1) and 2 (EA2).

	**PC**	**Model**	**Nugget effect**	**Sill (C1)**	**Range**	**RMSE**	**R^2^**	**MSDR**	**SDI (%)**	**SDI Clas.**
EA1	PC_1_	gau	0.0000	7.7255	32.2019	1.9733	0.4305	0.9801	8.4280	Weak
	PC_2_	sph	1.3378	1.1662	193.0948	1.3304	0.3131	0.9923	17.5121	Strong
EA2	PC_1_	exp	0.0000	4.5726	6.6577	2.1537	0.5986	0.9999	1.0024	Weak
	PC_2_	exp	1.4913	182.9979	19881.5906	1.5167	0.2075	1.0602	2969.3416	Strong

*PC, principal component; PC1, principal component 1; PC2, principal component 2; Exp model, exponential; Sph model, spherical; Gau model, Gaussian; RMSE, root mean square error; R^2^, coefficient of determination; MSDR, mean square deviation coefficient; SDI, spatial dependence index (Seidel and de Oliveira, 2014); and SDI Class, classification of spatial dependence (Seidel and de Oliveira, 2016).*

**FIGURE 1:**
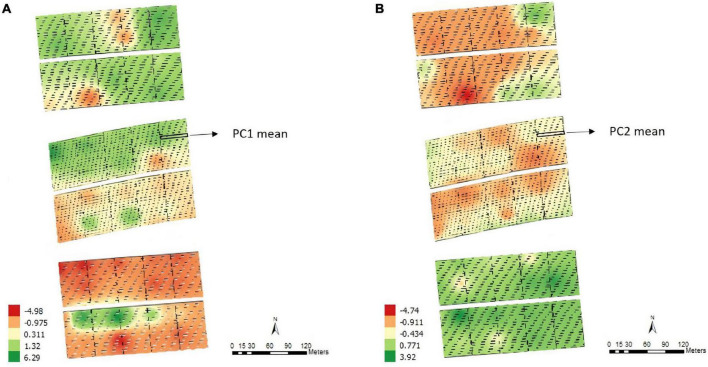
Thematic map generated by inverse distance weighting (IDW) and overlap of the experimental plots on the interpolated map and the average values of the points of interest for principal component 1 – PC1 **(A)** and principal component 2 – PC2 **(B)**, Experimental Area 1.

According to the map obtained by applying the IDW interpolation method (Figure 1), different variability patterns were observed for PC1 (Figure 1A). Negative values tended to represent regions where some parameters of PC1 had high positive expression, such as on the south side of the experiment. In this case, the area was most likely to have high levels of base saturation, calcium, magnesium, percentage base saturation, percentage aluminum saturation, aluminum, and potential acidity. Similar results were obtained when considering PC2 (Figure 1B), which was most associated with pH, phosphorus, silt and the contents of different fractions of sand. Each plot’s means for PC1 and PC2 values were obtained before proceeding with the subsequent analyses.

**FIGURE 2:**
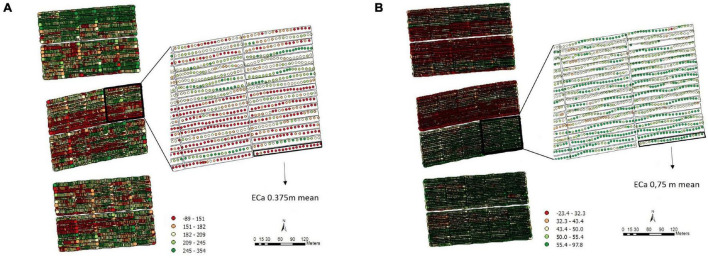
Apparent electrical conductivity (ECa) map and overlap of the experimental plots on the ECa map with 11,722 reading points and the average values of the points of interest in the 0.375 m **(A)** and 0.75 m **(B)** layers, Experimental Area 1.

For the soil ECa, high-density data were obtained (11,722 reading points), which eliminated the need for interpolation. We used the mean value of ECa at the two depths (0.375 and 0.75 m) in each plot for the analyses (Figures 2A,B). Moreover, it is possible to note different patterns of variability, which shows that the soil is not uniform throughout the fields.

**FIGURE 3:**
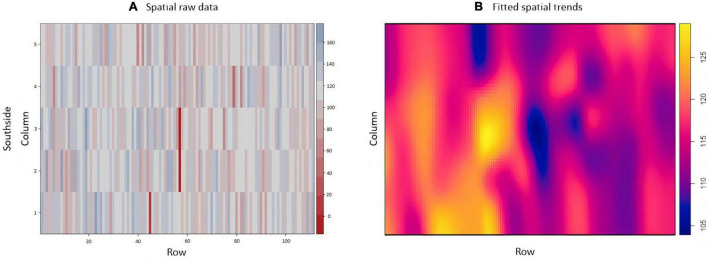
**(A)** Values of the raw data of tons of cane per hectare (TCH, tons/ha) for each experimental plot (family). **(B)** Visualization of the spatial pattern of the TCH values, Experimental Area 1.

The exploratory analysis of the raw TCH data from EA1 indicated variations in the TCH values for the different experimental plots (families) throughout the experiment (Figure 3A). The highest yields are shown in dark blue (maximum = 160 tons/ha), and the lowest yields are indicated in red (minimum = 0 tons/ha). Only three experimental plots showed null results due to data loss (dark red). Figure 3B details the spatial patterns; for example, the yellowish regions located on the south side indicated higher productivity, whereas the bluish concentrated areas on the north side showed lower productivity, thus indicating a spatial variability tendency.

Considering the inclusion of environmental covariates (in models 1.2 and 1.4) in the genetic-statistical models, only PC1 was included using forward selection because, in the first round, it presented the highest significance (*p* = 0.011) (Supplementary Table 1). In the second round, no variable was significant (Supplementary Table 2).

When considering the four models, some insights can be highlighted. First, the models that took into account the spatial dependence between rows and columns (1.3 and 1.4) allowed better results than the models that considered only a homogeneous variance over plots (1.1 and 1.2). For example, models 1.3 and 1.4 showed the highest H^2^ (0.60 and 0.62, respectively), the highest AC (0.77 and 0.79, respectively), the lowest CVe% (12.44 for model 1.3; 12.11 for model 1.4) (Table 2), and the lowest values of the AIC (4817.24 for model 1.3; 4810.63 for model 1.4) and BIC (4838.76 for model 1.3; 4822.14 for model 1.4) (Supplementary Table 3). Second, the inclusion of environmental covariates was similar for both models (1.2 and 1.4), i.e., the environmental effects (CVe%) were slightly reduced (13.40 for model 1.2; 12.11 for model 1.4). When considering the four models, the complete model (1.4) exhibited the best structure due to its capacity to model the H^2^ environmental effects and increase the AC estimates (Table 2). When considering the different VCOV structures for EA1, the lowest values of the AIC and BIC (Akaike, 1974; Schwarz, 1978) were obtained for model 1.4 (Supplementary Table 3). Therefore, this model is the most recommended model for predicting the genotypic values of the study population.

**TABLE 2:** Estimates of the variance components (REML) considering the different statistical models, Experimental Area 1.

**Model**	**TCH Mean**	**V_e_**	**V_g_**	**V_f_**	**CV_e_ (%)**	**CV_g_ (%)**	**CV_f_ (%)**	*H* ^**2**^	**AC**
1.1	119.53	264.20	125.30	389.50	13.60	9.40	16.50	0.49	0.70
1.2	119.65	256.60	132.50	289.10	13.40	9.62	14.21	0.51	0.71
1.3	120.20	223.69	165.50	389.19	12.44	10.70	16.41	0.60	0.77
1.4	120.12	211.60	175.30	386.90	12.11	11.02	16.36	0.62	0.79

*1.1: identity (ID); 1.2: identity including the significant covariate PC1 (ID + PC1); 1.3: first-order autoregressive structure (AR1 × AR1); and 1.4: first-order autoregressive structure including the significant covariate PC1 (AR1 × AR1 + PC1). The main characteristic considered was tons of cane per hectare (TCH), Experimental Area 1.*

*Ve, environmental variance; Vg, genetic variance; Vf, phenotypic variance; CVe (%), coefficient of environmental variation; CVg (%), coefficient of total genetic variation; CVf (%), coefficient of phenotypic variation;*H*^2^, broad-sense heritability at the average family level; and AC, accuracy of selection.*

### Exploratory Analysis of Data From EA2

The PCA performed with the 17 physical and chemical variables of the soil resulted in 44.1% of the variation being explained by the two first principal components (Supplementary Figure 4A). PC1 was essentially defined by the high positive values of aluminum saturation, aluminum, and clay variables, in contrast to the high negative values for the base saturation, calcium, percentage of base saturation, magnesium, pH, silt and cation exchange capacity variables. PC2 was mainly explained by the high positive values of the fine sand, percentage of base saturation, organic matter and pH variables, in contrast to the high negative values of the coarse sand, potential acidity and capacity to exchange cation variables (Supplementary Figure 4B). In contrast to what was observed in EA1, in this field, there was no clear division of the types of soil variables between PCs 1 and 2.

For EA2, the theoretical model of the semivariogram with the best fit to the data set was exponential for both PCs (Table 1). As observed for EA1, PC1 showed weak spatial dependence and PC2 showed strong spatial dependence. These results show that PC1 tends to present the soil variability in short-range distances, while PC2 often represents the long-range distance variability.

**FIGURE 4:**
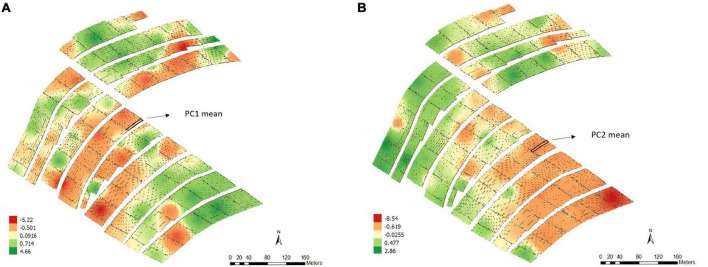
Thematic map generated by inverse distance weighting (IDW) and overlap of the experimental plots on the interpolated map and the average values of the points of interest for principal component 1 – PC1 **(A)** and principal component 2 – PC2 **(B)**, Experimental Area 2.

According to the map obtained by applying the IDW interpolation method (Figure 4), different variability patterns were observed for PC1 and PC2 (Figures 4A,B); i.e., the variability patterns are more likely to be associated with the previously cited variables.

**FIGURE 5:**
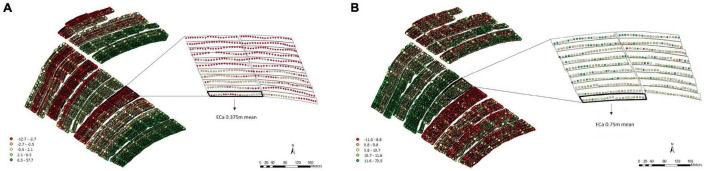
Apparent electrical conductivity (ECa) map and overlap of the experimental plots on the ECa map with 16,617 reading points and the average values of the points of interest in the 0.375 m **(A)** and 0.75 m **(B)** layers, Experimental Area 2.

For the soil ECa, 16,617 reading points were obtained within EA2 at depths of both 0.375 and 0.75 m (Figure 5). According to the data obtained for both depths, different patterns of variability were observed (Figures 5A,B).

**FIGURE 6:**
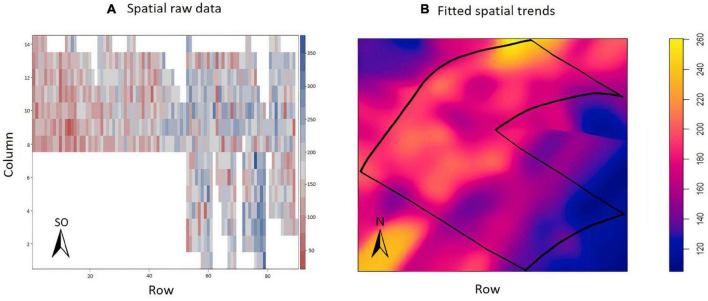
**(A)** Values of the raw data of tons of cane per hectare (TCH, tons/ha) for each experimental plot (family). **(B)** Visualization of the spatial pattern of the TCH values, Experimental Area 2.

The exploratory analysis suggested the presence of variations in the TCH throughout the experimental area (Figure 6A); the highest yields are indicated by the dark blue color (maximum = 350 tons/ha), and the smallest yields are indicated by the reddish color (minimum = 50 tons/ha). Sugarcane presented a higher yield potential in this field than in EA1. In this experiment, there were no missing data. Figure 6B details the spatial patterns.

When considering forward variable selection for genetic modeling, only the covariate ECa at 0.375 m (ECa05) was included in models 1.2 and 1.4, because in the first round, it presented a significance level below 0.05 (0.001 and 0.002, respectively); the other variables showed non-significant Pearson’s chi-square values (Supplementary Table 4). In the second round, the ECa1 variable became explanatory for both models, presenting chi-square values of 0.031 and 0.011 (Supplementary Table 5); the other variables were not explanatory, with values higher than 0.05.

The estimates of the variance components and genetic parameters of models 1.1, 1.2, 1.3, and 1.4 are shown in Table 3. For all models, the CVe presented values between 10 and 22%. A significant decrease was obtained when model 1.3 was considered. In addition, the inclusion of both covariates in this model (model 1.4) allowed for an expressive reduction in this parameter (CVe = 10.66%). For the total genetic variation coefficient (CVg), all models showed results above 10% for TCH. Nevertheless, the significant increase in this parameter was highlighted when models 1.3 and 1.4 were considered (Table 3). The broad-sense heritability increased for TCH when models 1.3 and 1.4 were considered (0.72 and 0.76, respectively). For the latter model, the highest accuracy of the selection value (AC = 0.87) was also observed (Table 3). Considering the different VCOV structures, with or without environmental variables, the lowest values obtained for the AIC and BIC were estimated for model 1.4 (Supplementary Table 3). Therefore, this model is the recommended model for predicting the genotypic values of the study population.

**TABLE 3:** Estimates of the variance components (REML) considering the different statistical models, Experimental Area 2.

**Model**	**TCH Mean**	**V_e_**	**V_g_**	**V_f_**	**CV_e_ (%)**	**CV_g_ (%)**	**CV_f_ (%)**	*H* ^**2**^	**AC**
1.1	173.60	1379.00	371.00	1750.00	21.39	11.10	24.10	0.35	0.59
1.2	172.60	1340.00	359.00	1699.00	21.20	10.98	23.89	0.35	0.59
1.3	173.27	391.30	513.20	904.50	11.40	13.06	17.36	0.72	0.85
1.4	173.50	341.80	533.70	875.50	10.66	13.32	17.05	0.76	0.87

*1.1: identity (ID); 1.2: identity, including the significant covariate ECa of the soil at 0.375 m and 0.75 m (ID + ECa05 and ECa1); 1.3: first-order autoregressive structure (AR1 × AR1); and 1.4: first-order autoregressive structure, including the significant covariate ECa of the soil at 0.375 m and 0.75 m (AR1 × AR1 + ECa05 and ECa1).*

*The main characteristic considered was tons of cane per hectare (TCH), Experimental Area 2.*

*Ve, environmental variance; Vg, genetic variance; Vf, phenotypic variance; CVe (%), coefficient of environmental variation; CVg (%), coefficient of total genetic variation; CVf (%), coefficient of phenotypic variation;*H*^2^, broad-sense heritability at the average family level; and AC, accuracy of selection.*

## Discussion

Inferring genotypic values through phenotyping during the initial stages of plant breeding is challenging due to the high presence of non-genetic (or environmental) variations. The environmental variance can be split into micro and macro conditions. The microenvironment, or the residual, can be controlled by experimental design and statistical methodologies. For example, linear mixed models increase the analysis precision due to the flexibility in modeling the field trial source of variations (Cursi et al., 2020, 2021; Hoarau et al., 2021). Additionally, residuals can be controlled by collecting data from the field (electroconductivity, physical and chemical variables) to include in the statistical model. Here, when the modeled microenvironmental variation was considered, the genetic parameters were best estimated, i.e., the highest heritability, CVg%, and lowest CVe%. We stress that the presented genetic parameters are strong indicators to perform inferences about a given trial.

On the other hand, macroenvironmental conditions are usually examined during the final stages of a breeding program, where genotype by environmental interaction is detailed, to identify the best genotypes for different environmental conditions. However, in the early stages, this approach is impractical due to the lack of material for each genotype. Therefore, RIDESA/UFSCar divides the sugarcane genotypes into two contrasting areas, where EA1 represents a favorable environment and EA2 represents an adverse environment. Other environmental conditions can be obtained, but they usually rely on between them. Considering this contrast, we note that controlling residual variance is better suited for adverse experimental areas.

The data acquired in this work allowed some insights. The usage of PCA for the composition of a fertility index based on linear combinations of soil variables (principal components) collected at a high sample density for the investigation of the possible structure of spatial variability proved to be efficient for the EA1. Similar results were reported by Silva et al. (2010), where PCA provided interpretable components and correlated with different physical and chemical attributes of the soil. According to these same authors, this type of analysis, in association with geostatistics, enabled an assessment of the variability of different soil components.

In this study, although PC1 had a weak spatial dependence for EA1, according to the classification proposed by Seidel and de Oliveira (2016), it was the variable most associated with TCH when incorporated into the genetic-statistical model. The four models showed CVe values between 10 and 20%, classified as medium magnitude (Couto et al., 2013). A high CVe magnitude value is not desired because it is indicative of a low degree of experimental precision and may be associated with considerable environmental variability, i.e., non-controlled variation. CVe presented the lowest values when the experimental error term was modeled for the spatial dependence between errors (first-order autoregressive structure). According to Gilmour et al. (1997), applying a spatial model in experimentation is quite efficient and desirable, as it improves the experiment’s precision. Slight improvement in this model was also obtained with the inclusion of PC1 (model 1.4), allowing a better estimate of the genotypic values of the study population since the experimental error was reduced.

In general, to evaluate the experimental quality, several statistics should be considered beyond the CVe value, such as the total CVg, broad-sense heritability, and AC. These statistics are essential to effectively determine the genotypic value of the genetic material resulting from phenotypic evaluations (Resende and Duarte, 2007). Considering EA1, only models 1.3 and 1.4 showed CVg above 10%, indicating genetic variability in the population for exploitation (Cursi et al., 2020). Additionally, the models that account for the soil spatial variability pattern (models 1.3 and 1.4) showed high magnitude values (*H*^2^ above 0.60) (Barreto et al., 2021). The inclusion of PC1 (models 1.2 vs. 1.1 and 1.4 vs. 1.3) slightly increased the value of *H*^2^, showing the contribution of this variable in explaining the model. Statistical modeling of residuals and the inclusion of environmental covariates improved the heritability, indicating that a large part of the evaluated phenotypic variation may be attributed to the variation in the effects of the genotype, with limited environmental confusion (Leite et al., 2006). The AC values were also high for all the models (higher than 0.70). When PC1 was added to models 1.2 and 1.4, the AC values also increased (Table 2). Unlike the pattern observed for EA1, the variable that best fitted the genetic-statistical model for EA2 was the ECa readings for both depths. This variable showed correlations with the physical and chemical soil attributes in both experimental areas (Supplementary Figures 3, 4). This result suggests that ECa readings were able to capture another type of soil variability not measured by the soil analysis performed in this study.

James et al. (2012) used ECa measurements obtained from the mapping of all experimental plots to characterize the salinity pattern present in the experimental field; such characterization allowed the separation of the area into three blocks with different salinity patterns. Under conditions of high ECa (or high salinity), a reduction in the yield of the genotypes under investigation was observed in the study by James et al. (2012). To correct the different patterns of spatial variability present in the experimental field, these same authors employed the separable first-order autoregressive structure (AR1 × AR1) to include information from the ECa sensor as a covariate in the genetic-statistical model. This strategy allowed an improved understanding and identification of the information of interest, reducing the estimation bias. Similar results were obtained in this study, in which the residual variation coefficient (CVe) showed a significant reduction when the AR1 × AR1 model was considered (models 1.3 and 1.4). However, this reduction was even more accentuated with the inclusion of both covariates obtained from the ECa reading depths (0.375 and 0.75 m—included in model 1.4). Regarding CVg, all the models showed results above 10%. Nevertheless, the significant increase in this parameter is highlighted when considering the AR1 ×AR1 approach (models 1.3 and 1.4) and becomes even more evident with the inclusion of both ECa readings (model 1.4). This finding demonstrates that the different information obtained *via* ECa allows us to reduce the environmental effects and to exploit the genetic variability present in the population more efficiently.

As EA1 is in a more favorable production environment for the development of sugarcane, presenting a soil with a higher and more uniform clay content (clay content ranging from 630 to 650 g kg^–1^), PC1 that included variables more related to soil acidity and the presence of bases in relation to the CEC helped reduce the environmental effect in genetic modeling. In contrast, because EA2 is in a more restrictive production environment, i.e., with sandier soil (160–180 g kg^–1^ of clay) showing a low water retention capacity, ECa stood out in the genetic modeling, as sugarcane responds intensely to variations of this nature.

For Xu (2016), novel tools (such as those presented in this study) that aim to adjust spatial variability can be efficiently incorporated into other areas of plant breeding, e.g., prediction models in genomic selection and genotype x environment interaction studies. This same author proposed the concept of “envirotyping” as a third “typing” technology complemented by genotyping and phenotyping. In the future, the “envirotyping” concept will need to focus on experimental plots and individual plants with the development of high-performance and precision envirotyping platforms to integrate genotypic, phenotypic and environmental information, so that a high-quality breeding system can be established with high efficiency and accuracy (Crossa et al., 2021).

## Conclusion

1. The use of a high-density soil sampling procedure and ECa data for modeling the spatial variability during the statistical analysis was efficient and provided the best scenario for breeding programs.

2. Using principal components based on high-density soil sampling data allowed us to identify a part of the total variability in the data for EA1. Therefore, principal components can be efficient indexes for incorporation as covariates in genetic-statistical models because they reduce the experimental error (CVe).
3. ECa sensors can be highly recommended to adjust the spatial dependence present in the early stages of sugarcane breeding programs, mainly for those in sandy soil regions, as observed for EA2. In addition, this type of geotechnology can be widely employed in agronomic experimentation and the various areas of study that focus on plant breeding, e.g., experimentation, genomic selection, and genotype x environment interaction studies.

## Data Availability Statement

The original contributions presented in the study are included in the article/Supplementary Material, further inquiries can be directed to the corresponding author/s.

## Author Contributions

DC, RG, and LA designed the study with assistance from HH and DD. DC, RG, LA, and TB performed the statistical analyses. All authors contributed to drafting the manuscript and developing the final version.

## Conflict of Interest

The authors declare that the research was conducted in the absence of any commercial or financial relationships that could be construed as a potential conflict of interest.

## Publisher’s Note

All claims expressed in this article are solely those of the authors and do not necessarily represent those of their affiliated organizations, or those of the publisher, the editors and the reviewers. Any product that may be evaluated in this article, or claim that may be made by its manufacturer, is not guaranteed or endorsed by the publisher.
