# Supplementary material for: Novel Tools for Adjusting Spatial Variability in the Early Sugarcane Breeding Stage
Source: Front Plant Sci. 2021 Nov 18;12:749533. doi: 10.3389/fpls.2021.749533 (PMC8638809; doi:10.3389/fpls.2021.749533)
Supplement: Supplementary file 1 [file Data_Sheet_1.docx]

Supplementary Material

# Supplementary Figures and Tables

## Supplementary Figures

##
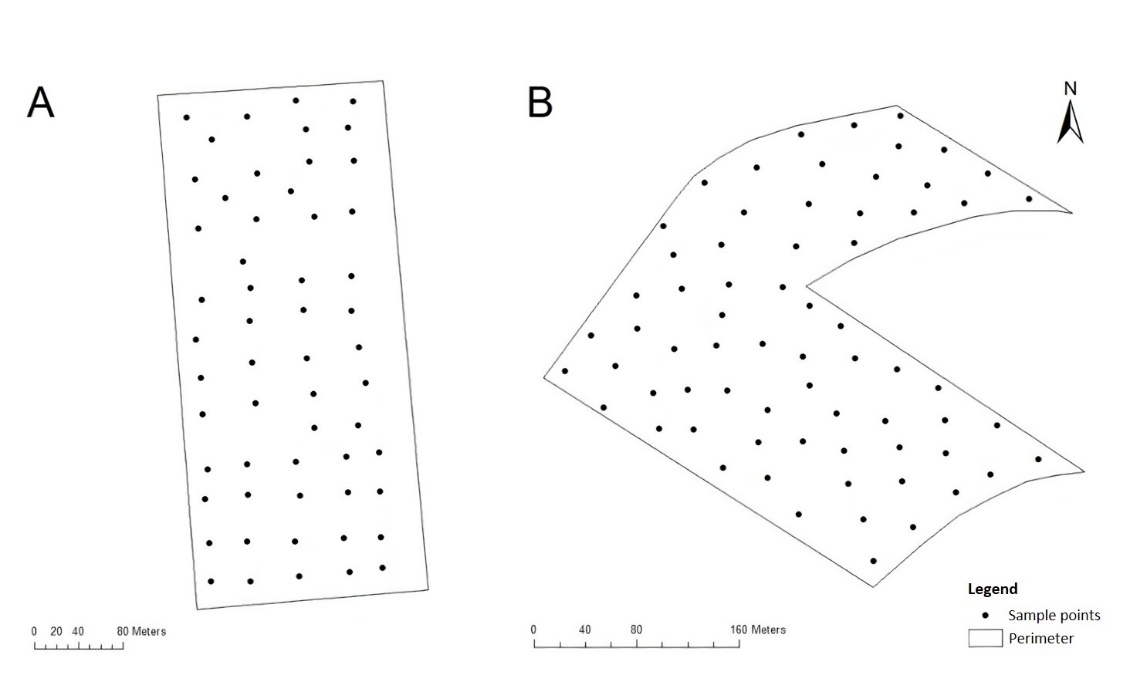


**Supplementary** **Figure 1.** (**A**) Experimental Area 1: 56 sample points in 6.5 ha; (**B**) Experimental Area 2: 68 sample points in 9.7 ha.


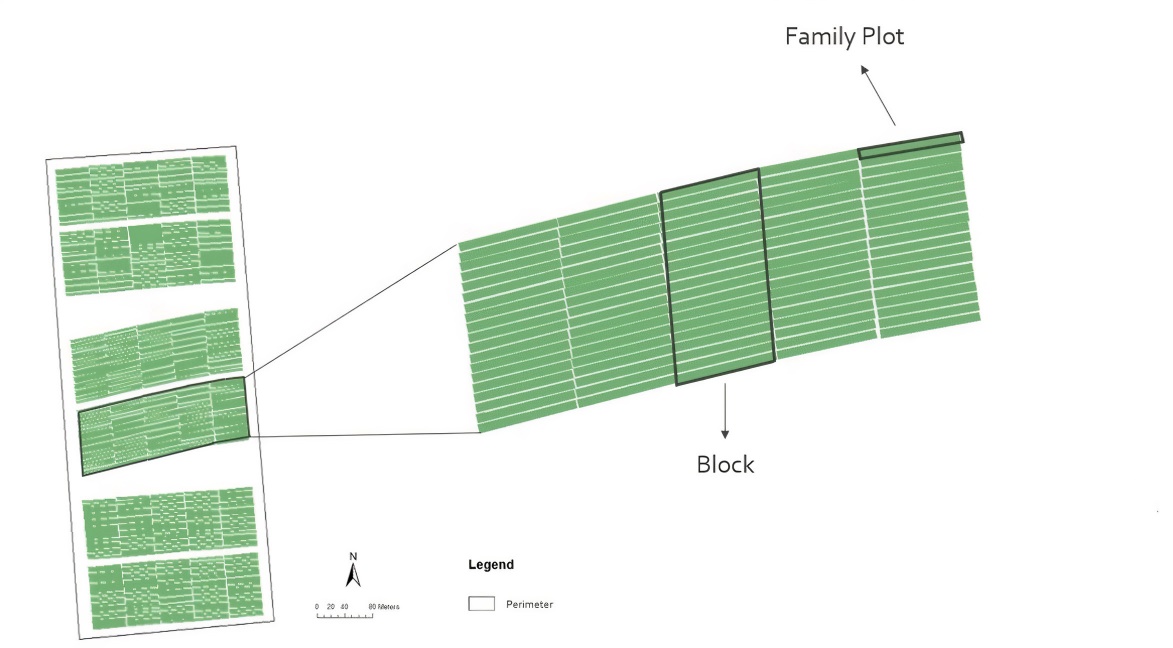


**Supplementary** **Figure 2.** Scheme of the position and location of each experimental plot to extract the average value of all of the pixels, Experimental Area 1. The same routine was enacted for Experimental Area 2.


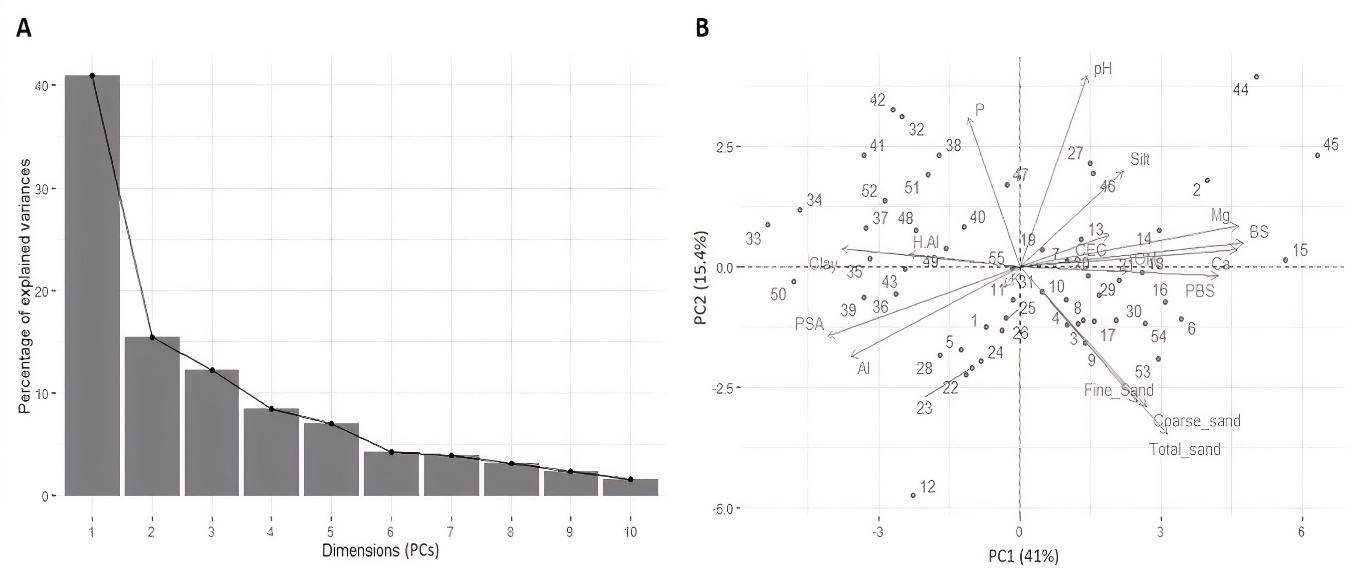


**Supplementary** **Figure 3.** (**A**) Scree plot of the eigenvalues of the principal components and (**B**) two-dimensional graph (biplot) of principal components 1 and 2 (PC1 and PC2) of the 17 physical and chemical variables from 55 soil sample points at depths of 0 to 20 cm in Experimental Area 1.


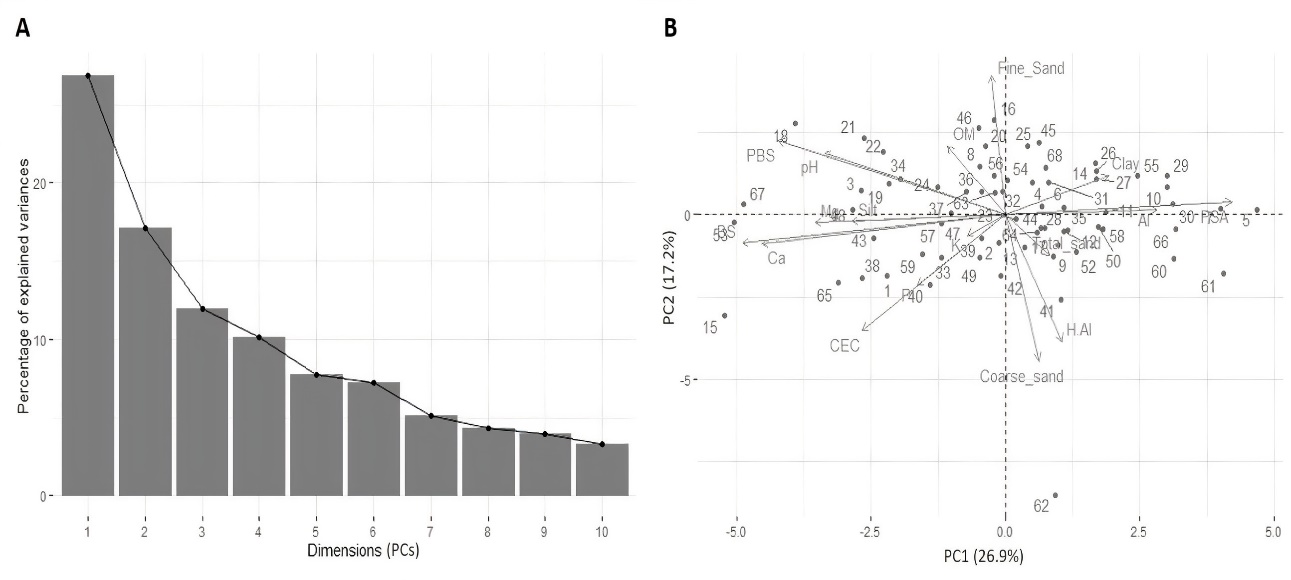


**Supplementary** **Figure 4**. (**A**) Scree plot of the eigenvalues of the principal components and (**B**) two-dimensional plot (biplot) of principal components 1 and 2 (PC1 and PC2) of the 17 physical and chemical soil variables from 68 soil sample points at depths of 0 to 20 cm in Experimental Area 2.

## Supplementary Tables

**Supplementary Table 1**. Covariates for the identity (ID) and first-order autoregressive (AR1 x AR1) models were selected using the forward method through the individual inclusion of different soil information and sensors. ECa05: apparent electrical conductivity of the soil at 0.375 m; ECa1: apparent electrical conductivity of the soil at 0.75 m; PC1: principal component 1; PC2: principal component 2. Information regarding Experimental Area 1.

| Model | Variable | Wald statistic | d.f. | p-value |
| --- | --- | --- | --- | --- |
| 1.1 (ID) | ECa05 | 0.35 | 1 | 0.555 |
|  | ECa1 | 0.01 | 1 | 0.930 |
|  | **PC_1_** | **6.49** | **1** | **0.011*** |
|  | PC_2_ | 0.72 | 1 | 0.398 |
| 1.3 (AR1 x AR1) | ECa05 | 0.24 | 1 | 0.626 |
|  | ECa1 | 0.02 | 1 | 0.877 |
|  | **PC_1_** | **6.34** | **1** | **0.012*** |
|  | PC_2_ | 1.10 | 1 | 0.295 |

* Significant variable considering a significance level of 0.05.

**Supplementary Table 2**. Inclusion of new variables for the identity (ID) and first-order autoregressive (AR1 x AR1) models when already considering PC1. ECa05: apparent electrical conductivity of the soil at 0.375 m; ECa1: apparent electrical conductivity of the soil at 0.75 m; PC1: principal component 1; PC2: principal component 2. Information regarding Experimental Area 1.

| Model | Variable | Wald statistic | d.f. | p-value |
| --- | --- | --- | --- | --- |
| 1.1 (ID) + PC1 | ECa05 | 0.12 | 1 | 0.728 |
|  | ECa1 | 0.06 | 1 | 0.812 |
|  | PC_2_ | 0.00 | 1 | 0.945 |
| 1.3 (AR1 x AR1) + PC1 | ECa05 | 0.09 | 1 | 0.759 |
|  | ECa1 | 0.06 | 1 | 0.807 |
|  | PC_2_ | 0.00 | 1 | 0.983 |

* Significant variable considering a significance level of 0.05.

**Supplementary Table 3**. Akaike information criterion (AIC) and Bayesian information criterion (BIC) values when considering the different VCOV structures, with the inclusion of significant covariables in the model, for the parameter tons of cane per hectare (TCH).

| Local | Trait | Model | AIC | BIC |
| --- | --- | --- | --- | --- |
| Experimental Area 1 | TCH | a) 1.1 | 4832.10 | 4840.71 |
|  |  | b) 1.2 | 4816.57 | 4829.48 |
|  |  | c) 1.3 | 4817.24 | 4838.76 |
|  |  | **d) 1.4** | **4810.63** | **4822.14** |
| Experimental Area 2 | TCH | a) 1.1 | 7328.67 | 7337.79 |
|  |  | b) 1.2 | 7301.28 | 7310.40 |
|  |  | c) 1.3 | 7300.72 | 7328.09 |
|  |  | **d) 1.4** | **7288.90** | **7306.16** |

ID: identity; AR1: first-order autoregressive structure; PC1: principal component 1; ECa05: apparent electrical conductivity of the soil at 0.375 m; ECa1: apparent electrical conductivity of the soil at 0.75 m.

**Supplementary Table 4**. Covariables were selected for the identity (ID) and first-order autoregressive (AR1 x AR1) models by using the forward method through the individual inclusion of different soil information and sensors. ECa05: apparent electrical conductivity of the soil at 0.375 m; ECa1: apparent electrical conductivity of the soil at 0.75 m; PC1: principal component 1; PC2: principal component 2. Information regarding Experimental Area 2.

| Model | Variable | Wald statistic | d.f. | p-value |
| --- | --- | --- | --- | --- |
| 1.1 (ID) | **ECa05** | **12.82** | **1** | **0.001*** |
|  | ECa1 | 0.51 | 1 | 0.474 |
|  | PC_1_ | 0.38 | 1 | 0.537 |
|  | PC_2_ | 1.56 | 1 | 0.212 |
| 1.3 (AR1 x AR1) | **ECa05** | **10.06** | **1** | **0.002*** |
|  | ECa1 | 0.00 | 1 | 0.976 |
|  | PC_1_ | 0.05 | 1 | 0.825 |
|  | PC_2_ | 2.85 | 1 | 0.092 |

*Significant variable considering a significance level of 0.05.

**Supplementary Table 5**. Inclusion of new variables for the first-order identity (ID) and autoregressive (AR1 x AR1) models when already considering the variable ECa05 (apparent electrical conductivity of the soil at 0.375 m). ECa1: apparent electrical conductivity of the soil at 0.75 m; PC1: principal component 1; PC2: principal component 2. Information regarding Experimental Area 2.

| Model | Variable | Wald statistic | d.f. | p-value |
| --- | --- | --- | --- | --- |
| 1.1 (ID) + ECa05 | **ECa1** | **4.63** | **1** | **0.031*** |
|  | PC_1_ | 0.19 | 1 | 0.665 |
|  | PC_2_ | 1.25 | 1 | 0.264 |
| 1.3 (AR1 x AR1) + ECa05 | **ECa1** | **6.48** | **1** | **0.011*** |
|  | PC_1_ | 0.07 | 1 | 0.791 |
|  | PC_2_ | 2.82 | 1 | 0.093 |

*Significant variable considering a significance level of 0.05.
